# The Effect of Poly (Ethylene glycol) Emulation on the Degradation of PLA/Starch Composites

**DOI:** 10.3390/polym13071019

**Published:** 2021-03-25

**Authors:** Sarieh Momeni, Erfan Rezvani Ghomi, Mohamadreza Shakiba, Saied Shafiei-Navid, Majid Abdouss, Ashkan Bigham, Fatemeh Khosravi, Zahed Ahmadi, Mehdi Faraji, Hamidreza Abdouss, Seeram Ramakrishna

**Affiliations:** 1 Department of Chemistry, Amirkabir University of Technology, Tehran 15875-4413, Iran; sara.momeni181@yahoo.com (S.M.); zahmadi@aut.ac.ir (Z.A.); 2 Center for Nanotechnology and Sustainability, Department of Mechanical Engineering, National University of Singapore, Singapore 117581, Singapore; fatemeh_khosravi22@yahoo.com; 3 Department of Organic Chemistry, Faculty of Chemistry, University of Mazandaran, Babolsar 47416-95447, Iran; s.shafiei@stu.umz.ac.ir; 4 Institute of Polymers, Composites and Biomaterials—National Research Council (IPCB-CNR), Viale J.F. Kennedy 54—Mostra d’Oltremare pad. 20, 80125 Naples, Italy; ashkan.bigham@ipcb.cnr.it; 5 School of Chemistry, College of Science, University of Tehran, Tehran 14155-6455, Iran; m4.faraji@gmail.com; 6 Department of Polymer, Amirkabir University of Technology, Tehran 15875-4413, Iran; hamidrezaabdouss@gmail.com

**Keywords:** polyethylene glycol, polylactic acid, starch mixture, hydrolytic degradation, biodegradation

## Abstract

As a hydrophilic renewable polymer, starch has been widely used in biocompatible plastics as a filler for more than two decades. The present study aimed at investigating the effects of polyethylene glycol (PEG), as a plasticizer, on the physicochemical properties of a hybrid composite—polylactic acid (PLA) and thermoplastic starch (TPS). A solvent evaporation process was adopted to gelatinize the starch and disparate PEG contents ranging from 3 to 15 wt.% (with respect to the sample weight) were examined. It was revealed that the increase in the PEG content was accompanied by an increment in the starch gelatinization degree. Referring to the microstructural analyses, the TPS/PLA mixture yielded a ductile hybrid composite with a fine morphology and a uniform phase. Nevertheless, two different solvents, including acetone and ethanol, were used to assess if they had any effect on the hybrid’s morphology, tensile strength and thermal properties. It was found that ethanol culminated in a porous hybrid composite with a finer morphology and better starch distribution in the PLA structure than acetone. As the result of PEG addition to the composite, the crystallinity and tensile strength were decreased, whereas the elongation increased. The hydrolytic degradation of samples was assessed under different pH and thermal conditions. Moreover, the microbial degradation of the PLA/TPS hybrid composite containing different PEG molar fractions was investigated in the soil for 45 days. The rate of degradation in both hydrolytic and biodegradation increased in the samples with a higher amount of PEG with ethanol solvent.

## 1. Introduction

The properties of polymers can be manipulated through an alteration in their chemical structure and preparation process [1]. The polymers are being applied in different areas such as packaging [2], automobile manufacturing [3], computer [4], clothing, etc., and also they are being used as additives in dyes, glues, coatings [5] and concrete [6]. Despite the fact that they are now an inseparable part of our lives, their low degradation rate makes those polymers a threat to the environment [7,8,9]. Degradation refers to any irreversible process—light, heat, moisture, chemical conditions and biological activity—leading to a change in the polymers molecular structure followed by influencing the physical and chemical properties [10,11,12].

Biodegradation is a phenomenon by which organic substances are cleaved by living organisms, followed by being degraded with oxygen (aerobic) or without oxygen (anaerobic) [13,14]. The biodegradation process is vital because it causes the removal of polymer products from the environment, or in other words, to return to the life cycle [15]. Microorganisms such as bacteria and fungi are involved in the degradation of natural and synthetic plastics [16]. The key parameters affecting the degradation kinetic are the polymer, organism type and environmental conditions. To penetrate a microorganism’s cell, most polymers are too large. Therefore, they need to depolymerize into smaller monomers to be uptaken and degraded [13,17]. The chemical structure is a decisive factor showing whether a polymer is biodegradable. There are some practical ways to promote biodegradation, including changing the polymers’ molecular structure and developing some microorganisms capable of consuming a particular carbon source [18].

The degradation process is caused in polymers when a change in their physical and/or chemical properties occurs [19]. Physical changes include reducing the molecular weight, tensile strength, elongation at break and teardrop in the level of transparency [20,21]. Chemical changes are referred to the alterations in the chemical structure of a material [22]. Taking sunlight as an example into account, the physical and chemical changes in polymers as the result of that show themselves as discolored cracks, loss of mechanical properties and loss of gloss [23]. Generally, when the degradation is caused by an optical source like sunlight, UV, etc., the polymer will be converted into a low molecular weight polymer followed by turning into carbon dioxide [24,25,26].

Biodegradable plastics can be referred to the thermoplastic materials such as polylactic acid (PLA), polycaprolactone (PCL), polyhydroxybutyrate (PHB), starch and polyvinyl alcohol (PVA) [27,28]. PLA is a linear aliphatic polyester obtained from renewable sources such as corn and sugar cane, making it an appropriate biological polymer [29]. The amounts of lactic acid entered into the human body through packaging are less than the lactic acid usually taken through eating food. Therefore, lactic acid-based polymers as environment-compatible materials are suitable alternatives for food packaging [30]. Considering the good processability of PLA, it has better potential than other biodegradable polymers such as PCL and it can be processed through injection molding, blow molding and thermoforming [31]. The diffusion of water, oxygen and carbon dioxide is weak through PLA [32]. The addition of an emulsifier to PLA increases the transient molecule’s solubility by increasing the free volumes and as the result, the toughness will be improved. To reach a biodegradable PLA with a desirable toughness, alloying with other biodegradation polymers is required, but it is costly [33].

To increase the biodegradability and decrease costs, PLA is often mixed with starch [34]. The PLA and starch mixtures are used in thermal products such as drinking cups, food trays, dishes and boxes, food packaging, children’s clothing and medical applications—dental implants, suture and bone screws [14,35]. Starch is a carbohydrate belonging to the polysaccharides family and it is composed of many glucose units. Starch has two molecular architectures: linear amylose molecules and branched amylopectin molecules [36]. Natural starch is not thermoplastic and has limited processability due to the large size of the particles (5–100 μm). In the presence of a plasticizer, thermoplasticity is provided at a high temperature and sheared/cut by overcoming the strong inter-molecular and intra-molecular hydrogen bonds of starch [37]. During this process called gelatinization, the melting temperature and glass transition temperature of starch decrease and become injectable, just like the traditional synthetic plastics [38,39]. It is worth mentioning that the branches with short chains decrease the crystallinity degree of aliphatic polyesters and the branches with long chains decrease the melting viscosity yielding a rigid starch [40,41]. Hence, the brittleness of PLA and starch mixtures is the main problem in most of the applications. A few plasticizers with low molecular weights, such as glycerol and sorbitol, are used in the mixtures [42]. Polyethylene glycol (PEG) is a common plasticizer and emulsifier used to reduce crystallinity and improve the mechanical properties of PLA/starch [43]. PEG is among the most applicable stabilizers and intermediates used in different industrial processes [44]. In addition, to decreasing the crystallinity degree, this plasticizer not only decreases Tm but also provides more thermal stability by producing sufficient interactions with the polymer’s structure and it is more efficient than other plasticizers—sorbitol, glycerol, etc. [45].

The present study aimed to investigate the physical, chemical and biological properties of PLA/TPS when different contents of PEG had been added. Under laboratory conditions, the degradation of PEG-added PLA/TPS was tested and tracked-in soil. The weight loss, molecular weight change and surface erosion were assessed. Moreover, the effects of time and surface morphology on the degradation rate were studied.

## 2. Materials and Methods

### 2.1. Materials

Granules of PLA (3 mm nominal granule size) and corn starch were purchased from Sigma-Aldrich (St. Louis, MO, USA). Corn starch was dried in an oven to remove excess moisture for 24 h at 70 °C before being used. Chloroform (CHCl_3_), acetone (C_3_H_6_O), PEG, ethanol, polyethylene (PE), phosphate buffer (PBS, pH = 7.4), sodium hydroxide (NaOH), hydrochloric acid 37 wt.% (HCl) were purchased from Merck, Darmstadt, Germany, and used without any further purification.

### 2.2. Film Preparation

The solution-casting technique was adopted to fabricate the films. Pure PLA (2.18 g) was dissolved in 30 mL of chloroform in a 150 mL flask. Then, the reaction mixture was magnetically stirred at a rate of 150 rpm. PLA was dissolved entirely in 2 h and starch (0.93 g) was added to the reaction mixture at ambient temperature. After dissolving the starch, a colorless solution was formed. The solution was degassed using ultrasonic irradiation for 2 h. Next, the solution was poured onto a glass plate and allowed to evaporate slowly at ambient temperature to form a film. The film was dried under vacuum to a constant weight and kept at room temperature for over a week to reach an equilibrium crystallinity [39].

Other samples were prepared with PEG as an emulsifier. These samples contained from 3 to 15 wt.% PEG (with respect to the sample weight) [46,47]. In brief and referring to the mentioned experimental procedure, the desired weight of PLA was dissolved in chloroform followed by pouring the dissolved starch into it. After obtaining a homogenous mixture, the reaction temperature was raised to 70 °C and then PEG in a 1:1 ratio mixture of acetone: chloroform (10 mL) was added to the reaction flask. Finally, the films were prepared the same as the procedure mentioned in the former paragraph. The thickness of films was 20–50 μm. The all-prepared composite samples are listed in Table 1.

The PSE_22_, PSE_32,_ PSE_42_ and PSE_52_ films were prepared similarly to the PSE_2_, PSE_3,_ PSE_4,_ and PSE_5_ and their compositions were identical, but ethanol just replaced acetone. The resulting samples were compared with the reference sample of PE and starch with the combination of 30% starch and 70% PE (PES) [48]. All the stages were performed on the reference samples as well.

### 2.3. Photodegradation of the Samples

The films were cut (5 cm × 1 cm) and placed in a UV cabinet up to 500 h. Then, the samples were taken out of the cabinet and their characteristics were investigated.

### 2.4. The Microstructures of the Samples

The scanning electron microscopy (SEM, AIS 2100, Uiwang-si, Korea) was applied to assess the microstructure of samples and also the surface of samples during the degradation period was observed to check if any changes occurred. It is important to notice that the micrographs were taken from the samples’ surfaces before and after landfilling. In the case of landfilled samples, after removing the soil, they were rinsed carefully and dried in a vacuum oven at 60 °C. The experiment was performed on the samples after the sixth week.

### 2.5. Mechanical Performance

The tensile strength of prepared films was tested through an Instron 5566 tensile machine (Norwood, MA, US) with a stretch rate of 10 mm/min at room temperature. Three identical samples were tested and the average value of 5 samples was reported with a standard deviation.

The results belonging to the change in the breakpoint length besides the tensile strength are known by most researchers as a key factor in determining the degradation rate of polymeric compounds [49]. The below Equations (1) and (2) are applied to reach such information as follows:

(1) $$\mathrm{Retained}\;\mathrm{elongation}\;\mathrm{at}\;\mathrm{break}\;(\varepsilon_{r})\;=\;\frac{\varepsilon_{t}}{\varepsilon_{0}}$$

(2) $$\mathrm{Retained}\;\mathrm{tensile}\;\mathrm{strength}\;(\sigma_{r})\;=\;\frac{\sigma_{t}\;}{\sigma_{0}}$$

In this respect, ε_t_ and ε_0_ are the change in the breakpoint length of samples with and without being exposed to UV at different time intervals, respectively. Similarly, σ_t_ and σ_0_ are the tensile strength of samples with and without being exposed to light at different time intervals.

To track the effect of degradation on the mechanical properties, the tensile strength of samples before and after the UV radiation was tested at different time periods at ambient temperature. For this purpose, all prepared films were cut into six pieces (5 cm × 1 cm) and each piece was placed under UV radiation up to 500 h and at each specific time interval, the tensile strength test was taken. The mechanical tests were accomplished on the samples without being exposed to UV radiation to make a better comparison.

### 2.6. Differential Scanning Calorimetry (DSC) Study

Thermodynamic behaviors and non-isothermal crystallinity of the samples were studied through DSC in the presence of nitrogen gas. The samples (15 mg) were placed into the container and the applied heat was set at a rate of 10 °C/min. After reaching to the final temperature (200 °C), the samples were kept for 2 min and then again cooled at the same rate down to the room temperature.

### 2.7. Fourier Transform Infrared Spectroscopy (FTIR) Study

Changes in the chemical structure of samples after being exposed to UV were measured by a Fourier transform infrared spectrophotometer (Thermo, Nocolet 8700, Waltham, MA, USA). The test was conducted according to the KBr pellets method and in the wavelength range of 4000–400 cm^−1^.

### 2.8. Hydrolytic Degradation of the Samples

The samples with a dimension of 3 cm × 3 cm were cut, dried and placed in the glass vials of PBS and distilled water (pH = 7.4 ± 0.2). They were then placed in an incubator at 25 °C (room temperature) and 40 °C for three weeks. After the selected immersion periods, the selected samples were collected and rinsed with distilled water several times. Afterward, the rinsed samples were put into the oven to dry. This hydrolytic degradation was assessed in the neutral pH; the hydrolytic degradation in acidic and alkaline mediums were also investigated. To this end, the net weight of samples before and after hydrolytic degradation was measured.

The samples with the same dimensions were placed into the glass vials of NaOH solution (pH = 13). They were taken out at specific time intervals, rinsed with distilled water just like the previous step and placed in an oven at 70 °C for 3 h. Thus, hydrolytic degradation was investigated at the water bath at temperatures of 23 °C and 40 °C for three weeks. The sample weights were then measured precisely and an acid environment was created with hydrochloric acid. Afterward, hydrolytic degradation was analyzed according to alkaline and acid environments.

The residual weight after hydrolytic degradation (Q) was calculated based on Equation (3) as follows:

(3) $$Q=\;\frac{W_{t}}{W_{0}}\times 100$$

W_0_ and W_t_ represent the samples’ initial weight (g) and the samples’ weight (g) after being degraded at each specific time, respectively.

To make a better understanding of the samples’ hydrolytic degradation, the hydrolytic degradation (R, %h) was defined according to the following Equation (4):

(4) $$R=\;\frac{Q_{0\;}-{\;Q}_{t}}{t}$$

Q_0_ and Q_t_ represent the weight of the samples before and after degradation at specific time periods.

### 2.9. Biodegradation of the Samples

In this test, the samples were landfilled beneath 10 cm gardening soil in glass vases. Some samples were placed into the soil before exposure to UV and the others were exposed to UV up to 500 h first, weighted and then landfilled horizontally. It should be noted that the samples were placed into an incubator at 40 °C. During the procedure, the water content was preserved at about 20%. The weight loss test evaluated the biodegradation rate of the samples. Every two weeks, the samples were removed from the soil, rinsed with distilled water and dried for 10 h at 70 °C; after weighing the samples, they were returned to the soil.

The rate of biodegradation (Q′) was calculated based on Equation (5) as follows:

(5) $$Q^{\prime }=\frac{W_{1}-W_{2}}{W_{1}}\times 100$$

In this respect, W_1_ and W_2_ (Equation (5)) are the weights of samples before and after landfilling at different time intervals, respectively.

## 3. Results and Discussion

### 3.1. Mechanical Properties Evaluation

Tensile strength is one of the most important criteria for degradable polymers, specifically in the case of optical–oxidative degradation [50]. The rapid decrease in elongation of polymers is a meaningful sign giving valuable information about polymers’ structure. If a polymer’s elongation decreases, the rupture and fragmentation rate will increase under the environmental stresses [51]. The effects of PEG addition on the mechanical properties—tensile strength and elongation at break—of samples are tabulated through Table 2. PSE samples showed higher tensile strength than the PES sample, indicating that polyester has higher tensile strength than PE [52].

The decrease in the tensile strength of PLA when starch was added could be attributed to the incomplete miscibility of both hydrophobic (PLA) and hydrophilic (starch) phases [53]. By incorporation of PEG as an emulsifier to PLA/starch composite, the elongation at break could be increased through the improvement of PLA and starch miscibility and stability [54]. Comparing the effect of both solvents, acetone and ethanol, it was observed that using ethanol instead of acetone as PEG solvent caused more homogeneous emulsifier distribution in the polymer’s structure, which was why here the increase in elongation was observed. This phenomenon could be attributed to better emulsifier’s effect on the films through the gelatinization of starch [55]. Among the PSE_2_, PSE_3_, PSE_4_ and PSE_5_ samples, PSE_3_ was the one with the highest elongation percentage and the strength percentage of PSE_2_ almost equaled PSE_4_. The PSE_52_, PSE_42_, PSE_32_ and PSE_22_ samples had the least tensile strength attributed to the solvent changing. The highest percentage of elongation belonged to the PSE_52_, which had the least tensile strength percentage.

**Figure 1:**
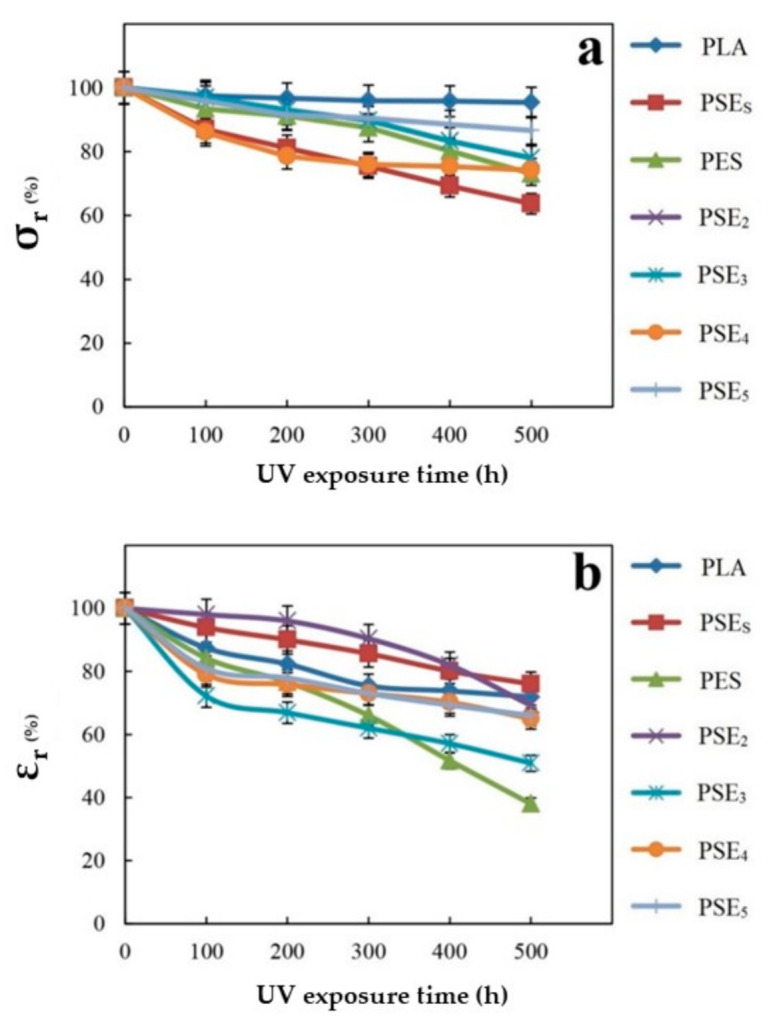
Changes of retained (**a**) tensile strength and (**b**) elongation to rupture point of polymer films after exposure to UV irradiation.

As the results showed better mechanical performance for acetone-based samples, those were selected to be evaluated after UV exposure. Figure 1 shows the effect of filler on the film’s tensile strength after exposure to UV light. According to Figure 1, the samples’ tensile strength dwindled from the beginning. The PSEs film experienced a sharper decrease than the other samples ascribed to the particle size and their distribution in the polymer’s matrix. The PSEs film sample showed a significant decrease in tensile properties after exposure to light and this reduction in tensile properties for PSE_2_, PSE_3_, PSE_4_ and PSE_5_ samples was less than PSE_s_. The reason could be attributed to the pores in the PSEs sample. These pores need the energy to get out of their normal size and stretched out. They must consume energy because of the particle’s smallness, but if the particle size is large, quite the opposite is the case and the tensile strength drops. There is a direct relationship between being exposed to UV irradiation and the decrease in samples’ mechanical properties [56]. The mechanical properties of the samples were declined sharply up to 500 h. The observed reduction in the mentioned properties was due to the degradation caused by UV irradiation. In the case of starch-containing samples, the reduction of tensile properties compared to the pure PLA films was due to the incompatibility of hydrophilic starch and hydrophobic PLA.

No significant change was observed in the tensile strength of PSE_3_ comparing to PSE_4_ and PSE_5_, but PSE_3_ showed the highest elongation at break among acetone-based samples. On the other hand, PSE_32_ exhibited the highest tensile strength among ethanol-based samples. Therefore, PSE_3_ and PSE_32_ are more acceptable and optimal composites for further investigations.

### 3.2. Morphology and Structural Properties

**Figure 2:**
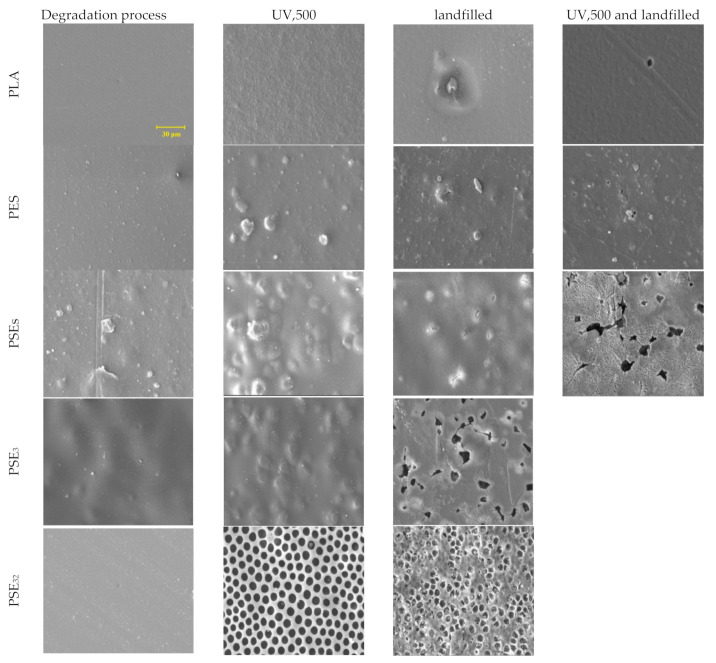
SEM micrographs of PLA film composites before and after degradation at 1.5 k× magnification.

The SEM was applied to study the samples’ surface morphology before and after landfills for up to two weeks, as shown in Figure 2. The SEM micrographs of the pure PLA sample show a flat and smooth surface. However, some scratches occurred on the PLA surface when UV irradiation up to 500 h was applied. It is clear that the polymer’s structure was degraded under UV light radiation and so the degradation process in the soil environment would become much more intense. According to the SEM results of PES, starch seeds were relatively well distributed in the structure. After being exposed to UV light for 500 h, the PES sample’s surface was affected negatively and the changes in the morphology are observable. As observed in the micrographs of PSE_s_, starch particles had acceptable distribution in the PLA phase, considering no emulsifier was used. PSE_s_ became sensitive after UV exposure, with some bubbles formed on the surface leading to a destruction in the surface after the landfill period. The PSE_3_ film micrograph before being treated with UV displayed a very smooth and uniform morphology with no pore or bubble on the surface. However, after exposure to UV light, the PSE_3_ film was mostly degraded and displayed some pores in the range of 1.5 to 2 µm on the surface.

After six weeks of landfilling, the SEM micrographs of samples showed progressive biodegradation for the PLA and starch samples (PSE_S,_ PSE_3_ and PSE_32_), whereas PES experienced less degradation than them. The reason might be found in the type of bonds in the PES sample, which is single covalent bonds [57]. Therefore, microorganisms showed less tendency towards the PES sample. The addition of starch to the PLA ester groups caused the compound more prone to degradation [58]. In contrast, it is known that the addition of polar groups to PE makes it more resistant to biological degradation [59]. Comparing the SEM images of PSE_3_ and PSE_32_ indicates the effect of PEG solvent on forming the pores in the film structure. As can be seen, the use of ethanol as a solvent resulted in better PEG dispersion and smoother surface in PSE_32_ [60]. It is important to notice that the UV irradiation caused a dramatic change in the films’ surface like forming bubbles and irregularities, leading to a faster degradation rate when they had been landfilled. It can be seen that the surface of samples after landfill became porous and also cracks were formed rooting in the microorganisms’ activity and their effect on the morphology. The degradation rate depends on different factors, among which the nature of microorganisms, soil humidity, pH, temperature and the physicochemical properties of the substrate can be enumerated [61]. The pure PLA sample did not experience a high degradation rate compared to the PLA/starch films. Therefore, it cannot be considered as a suitable biodegradable polymer. However, the addition of a biopolymer such as starch increased its biodegradability properties dramatically.

### 3.3. Thermal Behavior Assessment

**Figure 3:**
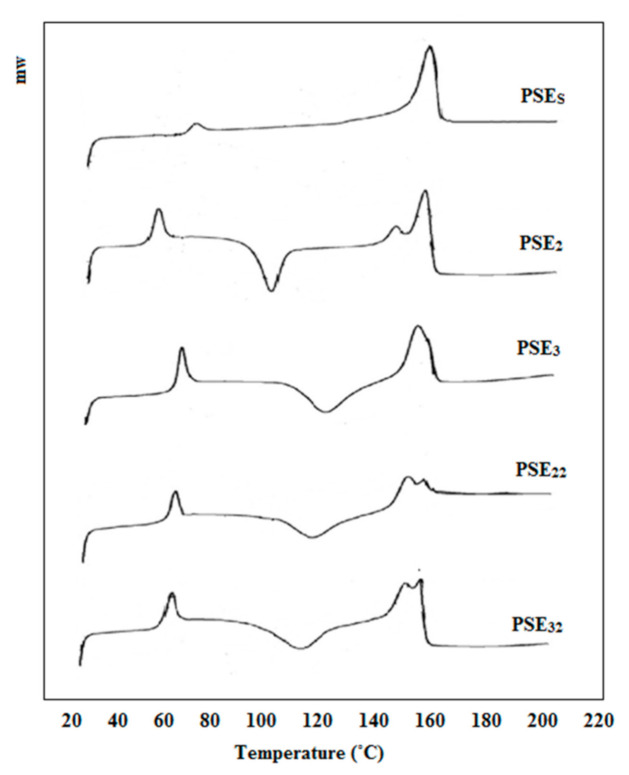
DSC curves of PSE_S_, PSE_2_, PSE_3_, PSE_22_ and PSE_32_ samples.

DSC studies were performed to investigate the effect of PEG addition on the PLA/starch composite. In addition, changes in the thermal properties of composites after using ethanol as the PEG solvent instead of acetone were observed. Figure 3 presents DSC analysis of PSE_S_, PSE_2_, PSE_3_, PSE_22_ and PSE_32_. In the case of PSE_2_, PSE_3_, PSE_22_ and PSE_32_ samples, there are three peaks visible—50–70, 100–120 and 140–160 °C. The first one (50–70 °C) pertained to the glass transition temperature (T_g_) followed by changes in the PEG phase. The second one (100–120 °C) is related to the crystallization temperature (T_c_) of PLA and the third one (140–160 °C) was attributed to the melting temperature (T_m_). As there is no significant change in the T_g_ and T_m_ in all samples, it can be concluded that the addition of PEG did not considerably affect the mobility of the polymeric chains [62]. A former study had shown that starch content in the PLA/starch blend had little effect on the T_m_ [63]. However, after the incorporation of PEG, T_g_ decreased slightly, which can be attributed to PEG’s emulsifier properties as well as its role as binding molecules between the hydrophobic (PLA) and the hydrophilic (starch) phases [64,65]. In the DSC curve of the PSE_s_ sample, two peaks are observable, referring to the T_g_ and T_m_ and the reason why no endothermic peak was seen pertained to using no emulsifier in this sample. The obtained results showed that PLA and starch were combined well through the solvent evaporation technique. The area under the T_g_ and T_m_ peaks gives information about the crystallinity percentage of samples. Therefore, it can be concluded that the addition of the emulsifier caused a decrease in the sample’s crystallinity, making the modified samples susceptible to a faster degradation [66]. With the addition of PEG to PLA/starch, the T_c_ peak appeared in the DSC diagram of PSE_2_. Then, the T_c_ of PSE_3_ increased with an increment in the content of PEG compared to PSE_2_, indicating an increase in the T_c_. These results are in accordance with SEM findings which showed that PEG-containing samples have faster degradation rates. After using ethanol instead of acetone as PEG solvent (PSE_22_ and PSE_32_), the peak at T_m_ was separated and reduced. This reduction in T_m_ intensity indicates a decrease in the crystallinity of the samples using ethanol solvent. It was also proved before that the amorphous structures show faster degradation rates [67]. This is again in accordance with SEM and mechanical properties results that ethanol-based samples showed higher degradation rates. On the other hand, the T_c_ was increased and the corresponding peak was broadened. These observations could be due to the uniformity of the composites and the better distribution of PEG after the application of ethanol as the solvent, which has increased the emulsifying effect of PEG on PLA/starch.

### 3.4. FTIR

**Figure 4:**
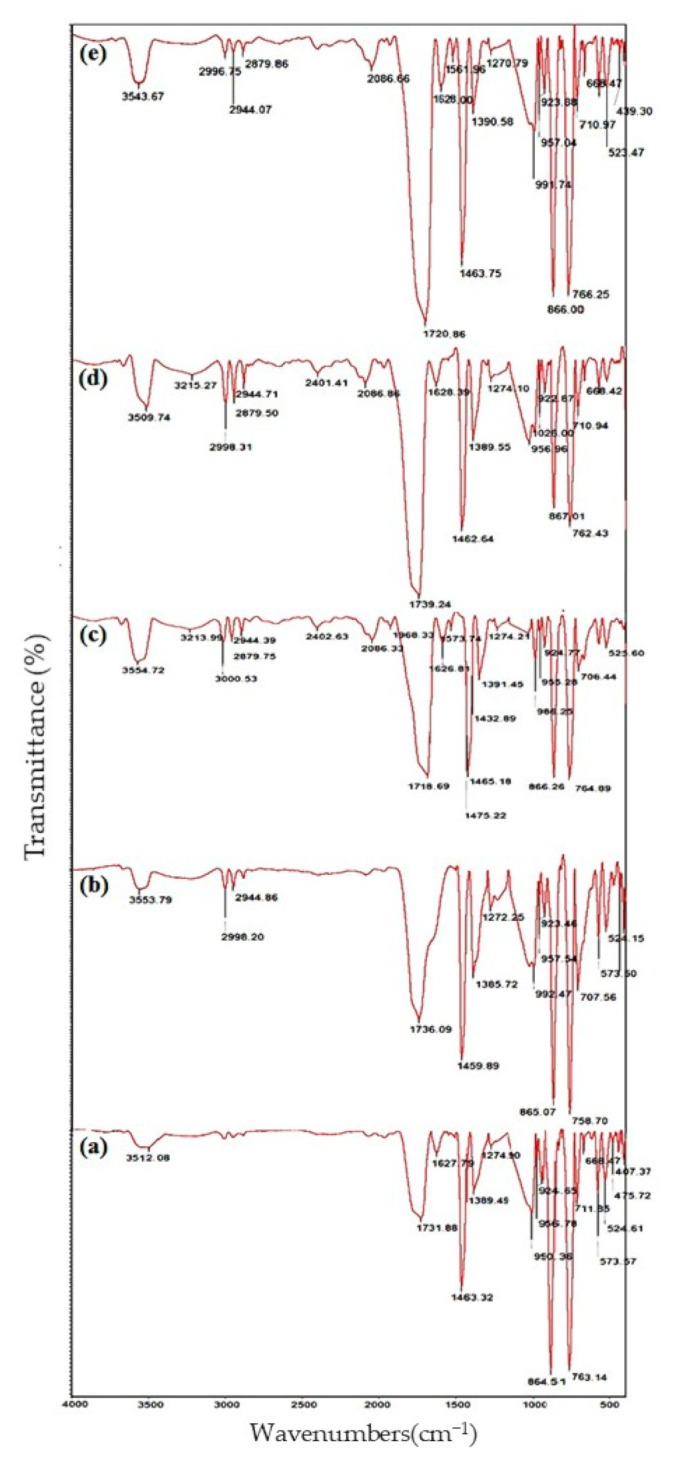
FTIR spectra of (**a**) PLA, (**b**) PSE_3_ and (**c**) PSE_32_, (**d**) PSE_3_,_500_ and (**e**) PSE_32,500_.

The FTIR was deployed to identify the chemical groups of hybrid composites including PLA (a), PSE_3_ (b), PSE_32_ (c), PSE_3500_ (d) and PSE_32,500_ (e) (Figure 4); through the taken analysis, the effects of both solvent and UV were assessed. The formation of oxygen-containing groups is a way by which the effect of UV on the chemical groups can be tracked [68]. The sample code PSE_3, 500_ signifies that the PSE_3_ sample was placed under UV radiation for 500 h. Changes in the carbonyl groups and the absorption changes were investigated through the band at 1716 cm^−1^. To reduce the error caused by the thickness of samples, the band at 1895 cm^−1^ was chosen corresponding to the CH groups as the reference; the absorption rate of each group was determined by dividing the absorption rate of that group to 1895 cm^−1^. The resulting films before and after the light seeing were taken at specified intervals of the FTIR spectra [47,69].

Optical-oxidative degradation of polymer samples is usually accompanied by the appearance of unsaturated bonds and various carbonyl groups by which the progression of degradation increases [70]. Two major observations—the formation of C=O (2000–1500 cm^−1^) and O-H groups in (4000–3000 cm^−1^) can also be found in the FTIR spectrum of polyolefin and other polymers [71].

When organic polymers are oxidized, carbonyl and hydroxyl groups are the predominant degradation products. These groups are easily characterized by an increase in bands’ intensity at 1710 cm^−1^ and 3400 cm^−1^. In the PLA spectrum, the band in the range of 3600–3500 cm^−1^ belonged to the C=O and the CH_3_ stretching vibrations were observed at 3000–2940 cm^−1^. The C=O stretching vibrations (1780–1761 cm^−1^) and C-O stretching vibrations (1190–1033 cm^−1^) were attributed to ester bonds. The broadband in the range of 3760–3010 cm^−1^ was attributed to the hydrogen bonds of starch hydroxyl groups. The stretching vibrations of CH_2_ and hydro glucose starch ring appeared in the range of 1180–960 cm^−1^ and 861–575 cm^−1^.

As can be seen in the PSE_3_ films FTIR spectrum, the absorption band related to the carbonyl group (1750–1640 cm^−1^) was more intense than PLA; this phenomenon could be attributed to the presence of optimal amounts of filler. On the other hand, with an increase in the amount of filler in the polymer matrix, the OH groups with reductive properties at the end of the chains were increased and, hence, it would partially prevent the polymer’s oxidation. By comparing the spectra of PSE_3_ before and after UV radiation, it is visible that prolonging the irradiation time was synchronized with an increase in the carbonyl absorption peaks; this phenomenon is an indicator of increasing in the oxidation rate of polymers [72]. Nonetheless, the effect of solvents—acetone and ethanol—on the chemical groups of samples was investigated. It is noteworthy that using ethanol instead of acetone did not cause the appearance of new peaks in the FTIR spectra, but a shift towards lower wavenumbers (1736 to 1718 cm^−1^). The possible phenomenon can be resulted from ethanol to increase the hydrogen bonds between PLA (carbonyl groups) and starch. This increment led to an increase in the bond length, followed by shifting the carbonyl peak towards lower wavenumbers [73]. Moreover, the FTIR peaks (oxygenated functional groups) of PSE_32,500_ experienced an increase in their intensities due to the better distribution of starch particles all over the structure.

### 3.5. Hydrolytic Degradation

**Figure 5:**
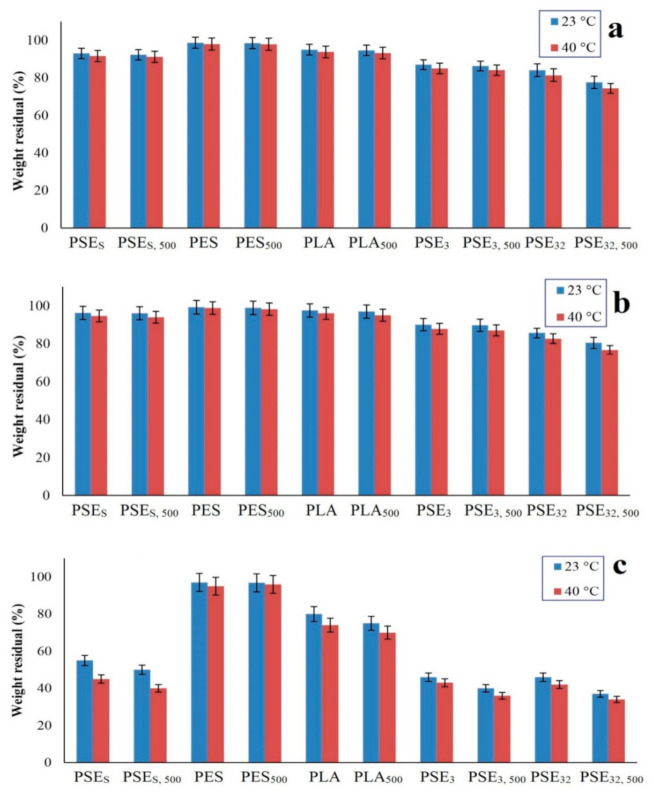
The weight residual after hydrolytic degradation of samples at (**a**) pH = 4, (**b**) pH = 7 and (**c**) pH = 13.

**Figure 6:**
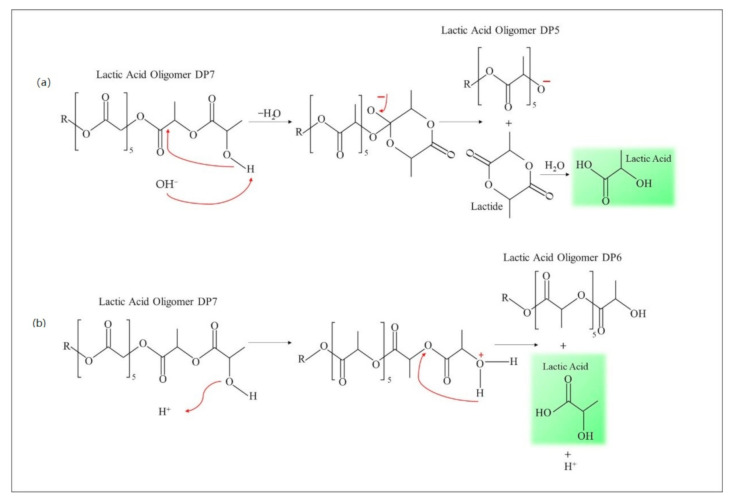
Hydrolytic degradation mechanism in (**a**) alkaline environment and (**b**) acidic environment.

Figure 5 shows the hydrolytic degradation percentage of samples at pH 4, 7 and 13 and two temperatures of 23 °C (room temperature) and 40 °C in order to evaluate the effect of pH and temperature increase on degradation rate. Considering Figure 5a, degradation kinetic is faster when the temperature is increased. The highest degradation at pH = 4 occurred for the PSE_32,500_ according to the mechanism shown in Figure 6b. Therefore, the time duration of being exposed to the light is effective in hydrolytic degradation. Another important parameter affecting the degradation results is the solvent, which showed higher degradation in the case of ethanol. Both the effect of UV exposure and the solvent was is in accordance with mechanical and SEM results. Thus, by increasing UV exposure duration, hydrolytic degradation of the samples is increased and hydrolytic degradation is similarly increased at higher temperatures. The lowest degradation belonged to the PES, indicating that PE has a low potential for hydrolytic degradation. The difference between PES and PSE_3_ is in the main polymeric phase in the samples due to the potential for hydrolysis [74,75].

The percentage of hydrolytic degradation at pH = 7 was less than pH = 4. It could be attributed to the concentration of H^+^ ions and their role in the hydrolysis of films [76]. As shown in Figure 5b, there was no significant difference between hydrolytic degradation percentages at both environments and the highest degradation was related to the PSE_32,500_, which experienced a higher hydrolytic degradation in 40 °C. The lowest hydrolytic degradation was related to PES having less degradation relative to the main sample of PSEs.

According to Figure 5c, the hydrolytic degradation percentage in alkaline environments (pH = 13) was more than the degradation percentage in neutral and acidic environments (pH = 4 and pH = 7). It can be due to the high hydrolysis power of OH^−^ ions which can hydrolyze polymer films at a high rate. Among the samples, PSE_32,500_ had the highest degradation percentage and the effect of UV radiation on film’s degradation was evident. Additionally, the lowest degradation percentage belonged to the PES sample. Based on the obtained results, the hydrolytic degradation of samples at pH = 13 occurred much faster than at pH = 4 and pH = 7. Making a comparison between Figure 5 revealed that the hydrolytic degradation of samples at 40 °C was more than that of 23 °C.

Figure 6 shows the mechanism of oligolactate degradation. The degradation in alkaline environments was accompanied by an intra-molecular ester exchange. The nucleophilic attack of the hydroxyl group to the second carbonyl causes the formation of a sustainable six-member interface loop and this reaction is the base of catalysis (mechanism a). According to this mechanism, oligomer lactic acid DP5 and lactic acid were produced during hydrolytic degradation. During the experiment, significant hydrolytic degradation was observed for polylactic/starch/PEG with acetone and PSEs samples which were almost fully degraded. The rupture of ester bonds in the hydroxyl groups of oligomers happens in low pH through protonation of the OH groups and through an intramolecular hydrogen bond [13]. A five-member ring may be one of the most stable intermediate structures. The formation of hydrogen bonds increases the electrophilic nature of carbonyl groups and hydrolysis through water molecules would occur through the site. According to this experiment, the degradation of various ester groups in lactic acid oligomers was independent of pH. Considering the low concentration of OH groups, the primary phase of these systems is stochastically degraded in polymer chains such that adding starch in polymer degradation increase hydrolytic degradation. Increasing starch in the matrix leads to an increase in hydrophilicity which can be attributed to the constant increase of dielectric. A higher dielectric constant leads to faster degradation and protecting the hydroxyl groups leads to a significant decrease in the hydrolytic degradation [77,78,79,80].

### 3.6. Biodegradation

**Figure 7:**
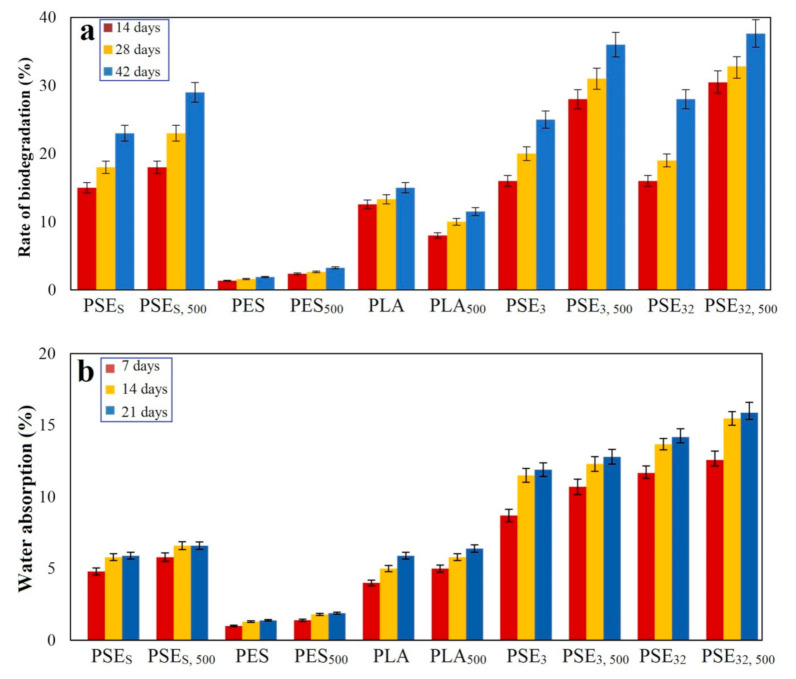
Polymer samples (**a**) rate of biodegradation and (**b**) water absorption after landfill at different times.

The biodegradation of samples was assessed during optical oxidative degradation. Due to the complexity of the biodegradation process and many factors affecting this process, various methods have been reported by researchers and some standards have been developed in this area [81,82,83]. However, applying them into practice often requires the use of special equipment. Biodegradation through the landfill is an easy way by which the degradation rate can be estimated [84]. The soil contains different microorganisms leading to the digestion of polymers followed by converting them into water and CO_2_ and subsequently the sample’s weight loss. The results are presented in Figure 7.

As can be observed in the case of control samples (PES), their weight loss was less than PLA samples. In addition, those samples, which were not in the exposure of UV, experienced less change in their weight during the period of the landfill than the samples treated with UV. The weight loss of samples was impressive, particularly after two weeks of landfill. The main mechanism governing this degradation process starts with the formation of pores in the polymers’ structure after being exposed to UV and so it leaves behind more surface area leading to an increase in the oxidation and digestion of substrates. Moreover, the breakage of polymer chains due to mechanical stresses is another parameter that may lead to the creation of active radicals and the oxidation of polymer in the environment.

The hydrophobic property of polyolefin is a preventive factor that impedes the penetration of enzymes into polymers’ structure and, thus, makes them useless. Moreover, many microorganisms and fungi require a suitable substrate besides a food source in order to proliferate and develop their colonies. Hence, polyolefin films are not suitable for the growth of microorganisms [39]. To make polyolefin biodegradable, their molecular mass must be dropped massively. Although environmental factors such as light, heat and oxygen are effective for this purpose, they cannot cause massive molecular mass drop within a relatively short period of time [85].

The addition of starch to the PLA structure would improve its wettability culminating in a faster water molecules penetration. The water absorption of samples is shown in Figure 7b, where the PSEs sample showed a higher absorption capability than the PES sample. Considering that no compatibilizer was used to prepare these samples, the PLA and starch compound was found to be mixed well with a proper distribution of starch through the whole structure. It is noteworthy that the PESs polymer film should have shown at least 20% water absorption, while only about 5% was obtained. The results are in accordance with SEM studies of composites (Figure 2). As can be seen from the SEM images, the highest degradation rate after the landfill is related to PSE_32_ due to the better PEG dispersion in the PLA/starch blend.

In the case of using ethanol as the solvent, higher water absorption in the prepared samples was observed. The reason for this phenomenon is rooted in the lack of compatibilizer, which can lead to bonding PLA and starch. Since these two are naturally hydrophobic (PLA) and hydrophilic (starch), their mixture would yield a physical bond even without using a compatibilizer leading to an increase in the compound’s water absorption rate. The maximum water absorption occurred in the first week, followed by a decrease in the rate up to the second week; this can be due to the saturated hydrophilic groups and saturated pores found in the compounds.

## 4. Conclusions

In summary, the emulsifier’s effect in the distribution and compatibility of thermoplastic starch and PLA phases containing the different percentages of PLA, Starch and emulsifier was investigated. According to the mechanical test and SEM results, the use of ethanol led to a better distribution of the PEG in the matrix and a higher degradation rate. In addition, FTIR results showed the effect of UV radiation on increasing the hydrophilicity of the samples and the degradation rate. The hydrolytic degradation of these samples was investigated under different pH and thermal conditions before and after UV irradiation. The results of the hydrolytic degradation of samples showed that the pH of the basic degradation rate was much higher than the acidic and neutral environments and by increasing the temperature, the degradation became more intense. Moreover, the PLA and starch films with varying PEG percentages were landfilled for 45 days and then studied by SEM. It showed some clear evidence of degradation, such as higher surface roughness, grooves, pores and decay. Various holes and grooves and a coordinating degradation of consortium microbial colonies, including fungi, bacteria and actionists, were observed on the film surfaces. The primary mechanism for biodegradation of polymers with a high molecular weight was oxidation or hydrolysis by enzymes which led to the creation of functional groups and improved the hydrophobic properties. The polymer main chains were then degraded and the samples showed lower molecular weight and mechanical properties. Therefore, it will be more prone to microbial uptake.

## Figures and Tables

**Table 1:** Composition of PLA/Starch/PEG samples.

Sample	PLA (wt.%)	PE (wt.%)	Starch (wt.%)	PEG (wt.%)	PEG Solvent	UV Exposure (h)
PES	–	70	30	–	–	–
PES_500_	–	70	30	–	–	500
PLA	100	–	–	–	–	–
PLA_500_	100	–	–	–	–	500
PSE_S_	70	–	30	–	–	–
PSE_S, 500_	70	–	30	–	–	500
PSE_1_	70	–	27	3	acetone	–
PSE_2_	70	–	24	6	acetone	–
PSE_3_	70	–	21	9	acetone	–
PSE_3, 500_	70	–	21	9	acetone	500
PSE_4_	70	–	18	12	acetone	–
PSE_5_	70	–	15	15	acetone	–
PSE_22_	70	–	24	6	ethanol	–
PSE_32_	70	–	21	9	ethanol	–
PSE_32,500_	70	–	21	9	ethanol	500
PSE_42_	70	–	18	12	ethanol	–
PES_52_	70	–	15	15	ethanol	–

**Table 2:** The elongation at break and tensile strength of samples without subjecting to UV.

Sample	Elongation at Break (%)	Tensile Strength (MPa)
PLA	9.44 ± 5.23	47.04 ± 11.32
PES	8.14 ± 1.02	25.13 ± 6.08
PSE_S_	5.44 ± 0.70	39.91 ± 7.89
PSE_2_	8.66 ± 0.65	26.32 ± 6.32
PSE_3_	12.00 ± 0.25	23.99 ± 6.92
PSE_4_	8.22 ± 0.23	24.05 ± 4.87
PSE_5_	9.09 ± 0.81	23.01 ± 5.65
PSE_22_	9.50 ± 1.22	13.04 ± 1.12
PSE_32_	14.94 ± 1.85	15.50 ± 1.15
PSE_42_	21.11 ± 4.44	9.02 ± 0.58
PSE_52_	23.94 ± 6.02	8.23 ± 0.51

## Data Availability

The data presented in this study are available on request from the corresponding author.

## References

[1] Shakiba M., Nabavi S.R., Emadi H., Faraji M. (2021). Development of a superhydrophilic nanofiber membrane for oil/water emulsion separation via modification of polyacrylonitrile/polyaniline composite. Polym. Adv. Technol..

[2] Cinelli P., Coltelli M.B., Signori F., Morganti P., Lazzeri A. (2019). Cosmetic packaging to save the environment: Future perspectives. Cosmetics.

[3] Sarvankar S.G., Yewale S.N. (2019). Additive Manufacturing in Automobile Industry. Int. J. Res. Aeronaut. Mech. Eng..

[4] AlSalhi M.S., Alam J., Dass L.A., Raja M. (2011). Recent advances in conjugated polymers for light emitting devices. Int. J. Mol. Sci..

[5] Rezvani Ghomi E., Esmaeely Neisiany R., Nouri Khorasani S., Dinari M., Ataei S., Koochaki M.S., Ramakrishna S. (2020). Development of an epoxy self-healing coating through the incorporation of acrylic acid-co-acrylamide copolymeric gel. Prog. Org. Coat..

[6] Wang F., Yang J., Cheng H., Wu J., Liang X. (2015). Study on mechanism of desorption behavior of saturated superabsorbent polymers in concrete. Aci Mater. J..

[7] Krzan A., Hemjinda S., Miertus S., Corti A., Chiellini E. (2006). Standardization and certification in the area of environmentally degradable plastics. Polym. Degrad. Stab..

[8] Rezvani Ghomi E., Khosravi F., Tahavori M.A., Ramakrishna S. (2021). Circular Economy: A Comparison Between the Case of Singapore and France. Mater. Circ. Econ..

[9] Foroughi F., Rezvani Ghomi E., Morshedi Dehaghi F., Borayek R., Ramakrishna S. (2021). A Review on the Life Cycle Assessment of Cellulose: From Properties to the Potential of Making It a Low Carbon Material. Materials.

[10] Göpferich A. (1996). Mechanisms of polymer degradation and erosion. Biomaterials.

[11] Gagliardi M., Lenarda P., Paggi M. (2017). A reaction-diffusion formulation to simulate EVA polymer degradation in environmental and accelerated ageing conditions. Sol. Energy Mater. Sol. Cells.

[12] Rezvani Ghomi E., Khosravi F., Mossayebi Z., Saedi Ardahaei A., Morshedi Dehaghi F., Khorasani M., Neisiany R.E., Das O., Marani A., Mensah R.A. (2020). The Flame Retardancy of Polyethylene Composites: From Fundamental Concepts to Nanocomposites. Molecules.

[13] Shah A.A., Hasan F., Hameed A., Ahmed S. (2008). Biological degradation of plastics: A comprehensive review. Biotechnol. Adv..

[14] Hakkarainen M. (2002). Aliphatic polyesters: Abiotic and biotic degradation and degradation products. Degradable Aliphatic Polyesters.

[15] Eubeler J.P., Zok S., Bernhard M., Knepper T.P. (2009). Environmental biodegradation of synthetic polymers I. Test methodologies and procedures. Trac Trends Anal. Chem..

[16] Kawai F. (2010). The biochemistry and molecular biology of xenobiotic polymer degradation by microorganisms. Biosci. Biotechnol. Biochem..

[17] Li K., Al-Rudainy B., Sun M., Wallberg O., Hulteberg C., Tunå P. (2019). Membrane Separation of the Base-Catalyzed Depolymerization of Black Liquor Retentate for Low-Molecular-Mass Compound Production. Membranes.

[18] Kinane J.A., Benakanakere M.R., Zhao J., Hosur K.B., Kinane D.F. (2012). Porphyromonas gingivalis influences actin degradation within epithelial cells during invasion and apoptosis. Cell. Microbiol..

[19] Shakiba M., Kakoei A., Jafari I., Rezvani Ghomi E., Kalaee M., Zarei D., Abdouss M., Shafiei-Navid S., Khosravi F., Ramakrishna S. (2021). Kinetic Modeling and Degradation Study of Liquid Polysulfide Resin-Clay Nanocomposite. Molecules.

[20] Houchin M., Topp E. (2009). Physical properties of PLGA films during polymer degradation. J. Appl. Polym. Sci..

[21] Wei B., Qi H., Zou J., Li H., Wang J., Xu B., Ma H. (2021). Degradation mechanism of amylopectin under ultrasonic irradiation. Food Hydrocoll..

[22] Beltrán-Sanahuja A., Casado-Coy N., Simó-Cabrera L., Sanz-Lázaro C. (2020). Monitoring polymer degradation under different conditions in the marine environment. Environ. Pollut..

[23] Moetazedian A., Gleadall A., Han X., Ekinci A., Mele E., Silberschmidt V.V. (2020). Mechanical performance of 3D printed polylactide during degradation. Addit. Manuf..

[24] Yazdan Mehr M., Bahrami A., van Driel W.D., Fan X., Davis J.L., Zhang G. (2020). Degradation of optical materials in solid-state lighting systems. Int. Mater. Rev..

[25] Yuan J., Ma J., Sun Y., Zhou T., Zhao Y., Yu F. (2020). Microbial degradation and other environmental aspects of microplastics/plastics. Sci. Total Environ..

[26] Lu T., Solis-Ramos E., Yi Y., Kumosa M. (2018). UV degradation model for polymers and polymer matrix composites. Polym. Degrad. Stab..

[27] Saalah S., Saallah S., Rajin M., Yaser A.Z. (2020). Management of Biodegradable Plastic Waste: A Review. Advances in Waste Processing Technology.

[28] Khosravi F., Nouri Khorasani S., Khalili S., Esmaeely Neisiany R., Rezvani Ghomi E., Ejeian F., Das O., Nasr-Esfahani M.H. (2020). Development of a Highly Proliferated Bilayer Coating on 316L Stainless Steel Implants. Polymers.

[29] Avinc O., Khoddami A. (2009). Overview of poly (lactic acid)(PLA) fibre. Fibre Chem..

[30] Winursito I. (2013). Biodegradabilitas Polikarboksilat Dari Asam Alginat dan Tapioka. J. Litbang Ind..

[31] Rasal R.M., Janorkar A.V., Hirt D.E. (2010). Poly (lactic acid) modifications. Prog. Polym. Sci..

[32] Drieskens M., Peeters R., Mullens J., Franco D., Lemstra P.J., Hristova-Bogaerds D.G. (2009). Structure versus properties relationship of poly (lactic acid). I. Effect of crystallinity on barrier properties. J. Polym. Sci. Part B: Polym. Phys..

[33] Jeong J., Ayyoob M., Kim J.-H., Nam S.W., Kim Y.J. (2019). In situ formation of PLA-grafted alkoxysilanes for toughening a biodegradable PLA stereocomplex thin film. RSC Adv..

[34] Sun Y., Lee D., Wang Y., Li S., Ying J., Liu X., Xu G., Gwon J., Wu Q. (2021). Thermal decomposition behavior of 3D printing filaments made of wood-filled polylactic acid/starch blend. J. Appl. Polym. Sci..

[35] Ghomi E.R., Khosravi F., Neisiany R.E., Singh S., Ramakrishna S. (2021). Future of additive manufacturing in healthcare. Curr. Opin. Biomed. Eng..

[36] Zou W., Yu L., Liu X., Chen L., Zhang X., Qiao D., Zhang R. (2012). Effects of amylose/amylopectin ratio on starch-based superabsorbent polymers. Carbohydr. Polym..

[37] Nafchi A.M., Moradpour M., Saeidi M., Alias A.K. (2013). Thermoplastic starches: Properties, challenges, and prospects. Starch Stärke.

[38] Huneault M.A., Li H. (2012). Preparation and properties of extruded thermoplastic starch/polymer blends. J. Appl. Polym. Sci..

[39] Rhim J.-W., Ng P.K.W. (2007). Natural Biopolymer-Based Nanocomposite Films for Packaging Applications. Crit. Rev. Food Sci. Nutr..

[40] Wang L., Jing X., Cheng H., Hu X., Yang L., Huang Y. (2012). Rheology and Crystallization of Long-Chain Branched Poly(l-lactide)s with Controlled Branch Length. Ind. Eng. Chem. Res..

[41] Nozue Y., Kawashima Y., Seno S., Nagamatsu T., Hosoda S., Berda E.B., Rojas G., Baughman T.W., Wagener K.B. (2011). Unusual Crystallization Behavior of Polyethylene Having Precisely Spaced Branches. Macromology.

[42] Esmaeili M., Pircheraghi G., Bagheri R., Altstädt V. (2019). Poly(lactic acid)/coplasticized thermoplastic starch blend: Effect of plasticizer migration on rheological and mechanical properties. Polym. Adv. Technol..

[43] Yu Y., Cheng Y., Ren J., Cao E., Fu X., Guo W. (2015). Plasticizing effect of poly(ethylene glycol)s with different molecular weights in poly(lactic acid)/starch blends. J. Appl. Polym. Sci..

[44] Yue H., Zhao Y., Ma X., Gong J. (2012). Ethylene glycol: Properties, synthesis, and applications. Chem. Soc. Rev..

[45] Jiang X., Jiang T., Zhang X., Dai H., Zhang X. (2012). Melt processing of poly(vinyl alcohol) through adding magnesium chloride hexahydrate and ethylene glycol as a complex plasticizer. Polym. Eng. Sci..

[46] Feng J., Yang G., Zhang S., Liu Q., Jafari S.M., McClements D.J. (2018). Fabrication and characterization of β-cypermethrin-loaded PLA microcapsules prepared by emulsion-solvent evaporation: Loading and release properties. Environ. Sci. Pollut. Res..

[47] Huneault M.A., Li H. (2007). Morphology and properties of compatibilized polylactide/thermoplastic starch blends. Polymers.

[48] Walker A.M., Tao Y., Torkelson J.M. (2007). Polyethylene/starch blends with enhanced oxygen barrier and mechanical properties: Effect of granule morphology damage by solid-state shear pulverization. Polymers.

[49] Lambert S., Sinclair C.J., Bradley E.L., Boxall A.B. (2013). Effects of environmental conditions on latex degradation in aquatic systems. Sci. Total. Environ..

[50] Sheela T., Bhajantri R., Ravindrachary V., Rathod S.G., Pujari P., Poojary B., Somashekar R. (2014). Effect of UV irradiation on optical, mechanical and microstructural properties of PVA/NaAlg blends. Radiat. Phys. Chem..

[51] Sionkowska A., Płanecka A., Lewandowska K., Michalska M. (2014). The influence of UV-irradiation on thermal and me-chanical properties of chitosan and silk fibroin mixtures. J. Photochem. Photobiol. B Biol..

[52] Wüst D.M., Meyer D.C., Favre P., Gerber C. (2006). Mechanical and Handling Properties of Braided Polyblend Polyethylene Sutures in Comparison to Braided Polyester and Monofilament Polydioxanone Sutures. Arthrosc. J. Arthrosc. Relat. Surg..

[53] Pukánszky B., Tüdős F. (1990). Miscibility and mechanical properties of polymer blends. Makromol. Chemie. Macromol. Symp..

[54] Sharma S., Singh A.A., Majumdar A., Butola B.S. (2019). Tailoring the mechanical and thermal properties of polylactic ac-id-based bionanocomposite films using halloysite nanotubes and polyethylene glycol by solvent casting process. J. Mater. Sci..

[55] Rogovina S.Z., Aleksanyan K.V., Loginova A.A., Ivanushkina N.E., Vladimirov L.V., Prut E.V., Berlin A.A. (2018). Influence of PEG on Mechanical Properties and Biodegradability of Composites Based on PLA and Starch. Starch Stärke.

[56] Signor A.W., VanLandingham M.R., Chin J.W. (2003). Effects of ultraviolet radiation exposure on vinyl ester resins: Charac-terization of chemical, physical and mechanical damage. Polym. Degrad. Stab..

[57] Whiteley K.S., Heggs T.G., Koch H., Mawer R.L., Immel W. (2000). Polyolefins. Ullmann’s Encycl. Ind. Chem..

[58] Acioli-Moura R., Sun X.S. (2008). Thermal degradation and physical aging of poly(lactic acid) and its blends with starch. Polym. Eng. Sci..

[59] Zhao C., Qin H., Gong F., Feng M., Zhang S., Yang M. (2005). Mechanical, thermal and flammability properties of polyeth-ylene/clay nanocomposites. Polym. Degrad. Stab..

[60] Chen J., Spear S.K., Huddleston J.G., Rogers R.D. (2005). Polyethylene glycol and solutions of polyethylene glycol as green reaction media. Green Chem..

[61] Walse C., Berg B., Sverdrup H. (1998). Review and synthesis of experimental data on organic matter decomposition with respect to the effect of temperature, moisture, and acidity. Environ. Rev..

[62] Buzarovska A., Grozdanov A. (2012). Biodegradable poly(L-lactic acid)/TiO_2_ nanocomposites: Thermal properties and degra-dation. J. Appl. Polym. Sci..

[63] Jang W.Y., Shin B.Y., Lee T.J., Narayan R. (2007). Thermal properties and morphology of biodegradable PLA/starch com-patibilized blends. J. Ind. Eng. Chem..

[64] Liu J., Jiang H., Chen L. (2012). Grafting of Glycidyl Methacrylate onto Poly(lactide) and Properties of PLA/Starch Blends Compatibilized by the Grafted Copolymer. J. Polym. Environ..

[65] Xiong Z., Li C., Ma S., Feng J., Yang Y., Zhang R., Zhu J. (2013). The properties of poly(lactic acid)/starch blends with a functionalized plant oil: Tung oil anhydride. Carbohydr. Polym..

[66] Pegoretti A., Fambri L., Migliaresi C. (1997). In vitro degradation of poly (L-lactic acid) fibers produced by melt spinning. J. Appl. Polym. Sci..

[67] Zhou Q., Xanthos M. (2008). Nanoclay and crystallinity effects on the hydrolytic degradation of polylactides. Polym. Degrad. Stab..

[68] Naderi M., Aghabararpour M., Najafi M., Motahari S. (2020). An investigation into resorcinol formaldehyde carbon aero-gel/epoxy coatings: Exploring mechanical properties, ultraviolet stability, and corrosion resistance. Polym. Compos..

[69] Wang X.-L., Yang K.-K., Wang Y.-Z. (2003). Properties of Starch Blends with Biodegradable Polymers. J. Macromol. Sci. Part C.

[70] Therias S., Rapp G., Masson C., Gardette J.-L. (2021). Limits of UV-light acceleration on the photooxidation of low-density polyethylene. Polym. Degrad. Stab..

[71] Gharehdashli A., Mortazavi S., Rashidi H. (2020). Photodegradation of low-density polyethylene with prooxidant and pho-tocatalyst. J. Appl. Polym. Sci..

[72] Rosu D., Rosu L., Cascaval C.N. (2009). IR-change and yellowing of polyurethane as a result of UV irradiation. Polym. Degrad. Stab..

[73] Nie B., Stutzman J., Xie A. (2005). A Vibrational Spectral Maker for Probing the Hydrogen-Bonding Status of Protonated Asp and Glu Residues. Biophys. J..

[74] ElSawy M.A., Kim K.-H., Park J.-W., Deep A. (2017). Hydrolytic degradation of polylactic acid (PLA) and its composites. Renew. Sustain. Energy Rev..

[75] Bos H.L., Meesters K.P.H., Conijn S.G., Corré W.J., Patel M.K. (2012). Accounting for the constrained availability of land: A comparison of bio-based ethanol, polyethylene, and PLA with regard to non-renewable energy use and land use. Biofuels, Bioprod. Biorefin..

[76] Xu L., Crawford K., Gorman C.B. (2011). Effects of Temperature and pH on the Degradation of Poly(lactic acid) Brushes. Macromol..

[77] Albertsson A.-C., Varma I.K. (2002). Aliphatic polyesters: Synthesis, properties and applications. Degradable Aliphatic Polyesters.

[78] Liu H., Zhang J. (2011). Research progress in toughening modification of poly(lactic acid). J. Polym. Sci. Part B Polym. Phys..

[79] Muroga S., Hikima Y., Ohshima M. (2018). Visualization of hydrolysis in polylactide using near-infrared hyperspectral imaging and chemometrics. J. Appl. Polym. Sci..

[80] Avérous L. (2004). Biodegradable Multiphase Systems Based on Plasticized Starch: A Review. J. Macromol. Sci. Part C.

[81] Li G., Sarazin P., Orts W.J., Imam S.H., Favis B.D. (2011). Biodegradation of Thermoplastic Starch and its Blends with Poly(lactic acid) and Polyethylene: Influence of Morphology. Macromol. Chem. Phys..

[82] Torres A.V., Zamudio-Flores P.B., Salgado-Delgado R., Bello-Pérez L.A. (2008). Biodegradation of low-density polyethylene-banana starch films. J. Appl. Polym. Sci..

[83] Panahi L., Gholizadeh M., Hajimohammadi R. (2019). Investigating the degradability of polyethylene using starch, oxo-material, and polylactic acid under the different environmental conditions. Asia-Pacific J. Chem. Eng..

[84] Da Silva A.P., Pereira M.D.P., Passador F.R., Montagna L.S. (2020). PLA/Coffee Grounds Composites: A Study of Photodegradation and Biodegradation in Soil. Macromol. Symp..

[85] Chamas A., Moon H., Zheng J., Qiu Y., Tabassum T., Jang J.H., Abu-Omar M.M., Scott S.L., Suh S. (2020). Degradation Rates of Plastics in the Environment. ACS Sustain. Chem. Eng..

